# The experiences of lay health workers trained in task-shifting psychological interventions: a qualitative systematic review

**DOI:** 10.1186/s13033-019-0320-9

**Published:** 2019-10-14

**Authors:** Ujala Shahmalak, Amy Blakemore, Mohammad W. Waheed, Waquas Waheed

**Affiliations:** 1 Division of Population Health, Health Services Research & Primary Care, School of Health Sciences, The University of Manchester, Williamson Building, Oxford Road, Manchester, M13 9PL UK; 2 Division of Nursing, Midwifery and Social Work, The University of Manchester, Manchester, UK; 3 School of Medicine, The University of Leicester, Leicester, UK

**Keywords:** Task-shifting, Psychological intervention, Lay health worker, Training, Experiences, Qualitative review

## Abstract

**Introduction:**

The prevalence of common mental disorders, such as depression and anxiety, is high and the demand for psychological interventions and talking therapies is increasing. In order to meet this need, it is necessary to explore alternative methods to deliver talking therapies. Training lay health workers (LHWs) to deliver psychological interventions might be one possible solution to address current gaps in service provision. A number of studies have successfully used this approach to deliver psychological interventions in order to meet the demand for mental health care. Despite increased interest in this area, the evidence has not been synthesised or systematically reviewed.

**Methods:**

Electronic databases (MEDLINE, EMBBASE, PsycINFO and CINHAL) were systematically searched to specifically capture studies on task-shifting psychological interventions for common mental disorders. Data were extracted on the experiences of the lay-workers on training and therapy delivery. Thematic analysis was used to analyse the data. Themes and subthemes of LHWs views on receiving training, barriers and facilitators to therapy delivery, factors required to become a successful therapist and the impact of training and therapy delivery on the therapists are described.

**Results:**

10 studies were eligible for inclusion. Key messages were: LHWs were satisfied with training but wanted more robust supervision; not enough time was given to training on understanding mental health problems; LHWs grew in confidence and this impacted on their personal relationships with others.

**Conclusion:**

This is the first review to explore LHWs experiences in training and therapy delivery by synthesising existing qualitative research. A number of key messages derived out of this review can help in further improving the quality of the training programmes and highlighting the benefits that are available for the LHW in delivering psychological interventions.

## Introduction

Common mental disorders (CMDs) refers to depression and a range of anxiety disorders which lead to significant decline in health and functioning [1]. Depression is the leading cause of disability worldwide, and is a major contributor to the overall global burden of disease [2]. In 2015, the proportion of the global population who had depression was estimated to be 4.4% (322 million) [2]. Depression is associated with a range of negative outcomes, including decreased physical and social functioning [3, 4], high health care utilisation [5], poor quality of life [6], and increased risk of mortality [7].

A number of psychological interventions such as cognitive behaviour therapy (CBT) have been found to be effective in reducing symptoms of depression [8–10] and anxiety [11, 12]. In 2006, the increasing access to psychological therapies (IAPT) service was developed in the United Kingdom (UK) to increase access to psychological treatments, and in particular CBT, for people with CMDs. The NHS long term plan now aims to increase the number of people with depression and anxiety disorders who can access talking therapies through IAPT to 1.9 million by 2023/24 [13]. However, IAPT services are already under immense pressure with approximately 1.4 million new referrals to IAPT care providers between 2017 and 2018 [14]. Between 2017 and 2018 a total of 23,686 mental health staff left the NHS [15], and estimated levels of burnout in IAPT workers are among the highest seen in the mental health workforce [16] resulting in growing waiting lists and increased unmet needs.

Considering the increase in demand for treatments and raised prevalence targets, it is necessary to explore alternative methods for delivering psychological interventions. Task-shifting to lay health workers (LHWs) is one such alternative. It is a process of delegation which involves shifting tasks, such as the delivery of psychological interventions for CMDs, from highly qualified health workers to workers with fewer qualifications and minimum training, in order to increase the coverage of health care and to use resources more efficiently [17]. LHWs are individuals carrying out functions related to health care delivery; trained in some way in the context of the intervention; and usually have no formal professional or paraprofessional certificated or degreed tertiary education [18].

Global recommendations and guidelines on task-shifting suggest the implementation of this approach as a way to strengthen and expand the healthcare workforce to increase access to services [19]. A number of studies have shown the effectiveness of task-shifting for a range of conditions such as, the delivery of antiretroviral therapy for HIV [20], with the approach now being used in the psychiatric field [21, 22]. Several trials have successfully used task-shifting to deliver psychological interventions for a variety of mental illnesses. A Cochrane review investigating the effect of non-specialist health workers on people with mental health conditions found that compared with usual care, interventions delivered by non-specialist health workers may increase the number of adults recovering from depression or anxiety 2 to 6 months after treatment [risk ratio (RR) 0.30, 95% confidence interval (CI) 0.14 to 0.64] [23].

Studies that have adopted a task-shifting approach have used a range of LHWs, from paraprofessionals already working within health systems, such as nurses, to lay women from within the community [24]. Furthermore, laypersons have been used in studies in low- and middle-income countries (LMICs) to deliver psychological interventions [25–27], as a solution to the shortage of fully trained health workers. Qualitative data have shown that participants prefer LHWs from the same community who share common socio-demographic characteristics as they are more accessible [28] and less intimidating than a formal service [29]. Furthermore, it avoids a formal mental health label and diagnosis, a reason that often leads people to avoid mental health services due to the stigma attached [30].

In LMICs, laypersons often come from more deprived socioeconomic backgrounds and have minimal formal education. Therefore, in-depth training, supervision and support from mental health professionals is required for task-shifting to be successful and to ensure high quality care [31]. Systematic reviews of qualitative studies have previously been conducted on task-shifting of psychological therapies [23, 32]. However, to date no review has been conducted on lay workers’ experiences on training. Qualitative research methods such as in-depth interviews and focus groups are used to answer questions about experience and meaning to gain a deeper understanding of a participant’s perspective in interventions such as task-shifting [33]. Therefore, the aim of our review is to systematically review the qualitative literature on the impact of training and delivery of psychological therapies by lay health workers (LHWs). This systematic review is important to help us understand the existing knowledge in this area and to direct future training and delivery of task-shifting programmes.

### Objectives

The aims of this review were to answer two primary questions:

1. How have studies explored the experiences of LHWs who have received training and delivered psychological therapies for CMD?
  - What are the types of studies?
  - What are the characteristics of the LHWs described in the papers?
  - What are the characteristics of training?
2. What does the evidence say about the LHWs’ experiences in training and delivering psychological therapies?
  - What were the LHWs own experiences of receiving training?
  - What were the barriers and facilitators to therapy delivery?
  - What factors are required to effectively train LHWS to deliver psychological interventions?
  - What was the impact of training and therapy delivery on the LHWs?

## Methods

### Eligibility criteria

This review aimed to identify all papers that explored the experiences of LHWs delivering psychological interventions for CMDs. Therefore, we did not restrict the search to one type of qualitative methodology or to articles published during a particular period of time. A ‘lay health worker’ was defined as any health worker carrying out functions related to health care delivery; trained in some way in the context of the intervention; and having no formal professional or paraprofessional certificated or degreed tertiary education [18].

The inclusion criteria for studies were: (a) available in English, (b) used qualitative data, (c) studies in which the LHW was a lay person with no mental health experience and (d) studies describing LHWs’ experiences of training and therapy delivery.

The exclusion criteria were: (a) studies evaluating the effectiveness of training LHWs that did not include any qualitative data, (b) studies focusing on the experiences of those receiving therapy and (c) studies involving participants with serious mental illnesses such as schizophrenia, bipolar disorder and other psychoses.

### Literature search strategy

We searched databases (CINAHL, Medline, Embase, PsycINFO) from inception until 13th March 2018. All the searches were exported to EndNote and duplicate references were reviewed and removed. Searches were conducted to specifically capture studies on task-shifting psychological interventions for CMDs. The search was adapted from another systematic review investigating non-specialist health worker interventions for the care of mental, neurological and substance abuse disorders [23]. Key terms including: lay, voluntary, untrained, non-professional, task shift, were combined with terms for CMDs, including: stress, common mental, anxiety and depression. To further limit the search to meet the aims of this review, we then combined the results of this search with criteria to specify qualitative methods. These included: qualitative, interview, focus group, content analysis, discourse, grounded theory, ethnograph. The full search strategy can be seen in Additional file 1.

### Data extraction

Titles and abstracts were screened by one researcher (US) and full papers of potentially relevant abstracts were obtained. A second researcher cross-checked and agreed the included and excluded papers (WW) and a final decision was made. Data were independently extracted by two reviewers (US and MWW) onto a standardised Excel spreadsheet. Data was collected on study details including intervention and participants, design and methods, as well as the author’s interpretations of their data. Data extraction followed the guidelines for meta-ethnography outlined by Noblit and Hare [34], whereby first order constructs defined as direct participant quotes were extracted. Therefore where possible we extracted data on LHWs self-reported experiences of training, supervision, and therapy delivery. However, in many cases there was insufficient primary data; in this instance we extracted second order constructs, defined as the authors’ interpretations of participants’ quotes expressed as themes, extracted from both the results and discussion sections of papers in order to capture all constructs. Therefore, here the second order constructs referred to the authors’ interpretations of the LHWs’ experiences of training. Data extraction rigour was enhanced by continuous discussion within the review team, as US and MWW independently extracted the data, whilst WW reviewed both sets of extraction for consistency. CASP (Critical Appraisal Skills Programme) tool was used for assessing the quality of included studies [35]. However, the quality of studies was not an inclusion criterion in this review. The decision to avoid quality as an inclusion criterion was in consideration of the different context in which qualitative studies are conducted [36].

### Thematic analysis

The thematic analysis approach to analysing qualitative data by Braun and Clarke [37] was used to aid thematic identification and summarisation of data from included studies. The aim of this review was to descriptively summarise evidence from qualitative studies exploring the impact of training and delivery of therapy on LHWs, therefore thematic analysis was appropriate.

In utilising the thematic analysis approach, we conducted, all six phases described by Braun and Clarke [37]: (1) becoming familiar with the data, (2) generating initial codes, (3) searching for themes, (4) reviewing themes, (5) defining themes and, (6) the write-up process. All included studies were read multiple times to facilitate understanding of the key concepts published in the studies. The analysis was undertaken in a multidisciplinary team (PhD student (US), Medical student (MWW) and a Psychiatrist (WW)). Papers were read and re-read by two reviewers (US and MWW) and the extracted data was then grouped into broad themes by the reviewers (US, MWW and WW).

Broad themes and subthemes were then refined though discussion between (US, MWW and WW) until consensus was reached. Once the themes had been broadly agreed, one reviewer (WW) read through the data in each of the themes checking that the interpretation of the data was correct and suggesting changes based on the original context of the studies.

The results are reported in line with PRISMA guidelines for systematic reviews. Our themes are presented in accordance with our review objectives as listed above.

## Results

**Fig. 1:**
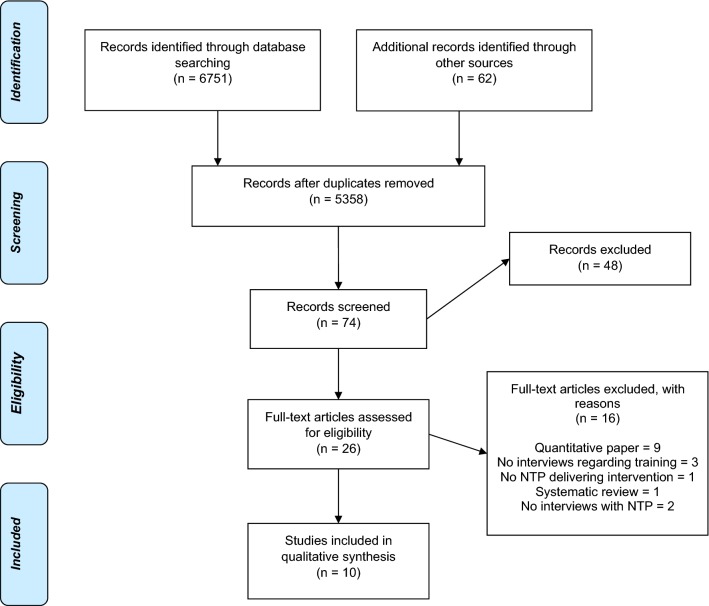
PRISMA flowchart representing the process of identifying relevant papers [38]

In summary, a total of 6751 papers were identified through the electronic search and a further 62 papers were identified through an electronic search which was conducted to find any systematic reviews that had investigated task-shifting. After duplicates were removed, 5358 were excluded after reading the title and a further 48 after reading the abstracts. 26 full text articles were screened for inclusion: 16 were excluded, resulting in a total of 10 articles to be included in this review. The flow diagram for the included papers can be seen in Fig. 1.

Characteristics of included studies. The 10 included studies were conducted in two high income countries (HICs) which were UK (n = 1) and Scotland (n = 1) and the remaining were conducted in LMICs, Pakistan (n = 4), India (n = 2), Nepal (n = 1) and Zimbabwe (n = 1). Six studies employed mixed methods and four studies were qualitative. Half of the studies used semi-structured interviews to collect data; two studies used focus groups, whereas one used both interviews and focus groups and another used open-ended questionnaires. A description of included studies is presented in Table 1.

**Table 1:** Table of characteristics of studies (n = 10)

First author/year	Participant type (no.)	Study design	Psychological intervention	Mental health condition	Data collection method	Method of analysis
Armstrong, J., 2003	Paraprofessional counsellors (12)	Mixed methods	Counselling	Unknown	Open-ended questionnaires	Content analysis
Naeem, S., 2003	Lay women (11)	Mixed methods	Counselling	Anxiety and depression	Focus groups and open-ended feedback	Unknown
Jordans, M.J., 2007	Paraprofessional counsellors (26)	Qualitative study	Counselling	Mild to severe psychosocial problems	Semi-structured interviews	Content analysis
Rahman, A., 2007	Lady Health Workers (24)	Mixed methods	Thinking Healthy Programme (CBT)	Perinatal depression	Focus groups	Systematic triangulation process
Pereira, B., 2011	Lay health counsellor (17)	Qualitative study	Psychoeducation	CMD	Qualitative semi-structured interviews	Thematic framework analysis
Coe, C., 2013	Volunteers befrienders (14)	Mixed methods	Perinatal support	Maternal anxiety and depression	Qualitative interviews	Unknown
Atif, N., 2016	Peer volunteers (8)	Qualitative study	Thinking Healthy Programme (CBT)	Perinatal depression	Interviews and focus groups	Thematic framework analysis
Maulik, P.K., 2016	Accredited social health activists (4)	Mixed methods	Mobile technology based electronic decision support system (EDSS)	CMD	Focus groups	Thematic analysis
Chibanda, D., 2017	Lay health workers (7)	Qualitative study	Problem solving therapy	CMD	Semi-structured interviews	Thematic content analysis
Khan, M.N., 2017	Lay helpers (2)	Mixed methods	Problem solving and behavioural activation	Anxiety and depression	Semi-structured interviews	Thematic content analysis

Two studies investigated the experiences of the LHWs in delivering the intervention [39, 40], whereas one study examined the barriers and facilitators of delivering the intervention through LHWs [41] and another examined the role of the voluntary sector in supporting women with perinatal mental health problems [42]. Two studies were conducted to investigate the perspective of stakeholders involved in the intervention [43, 44], whilst another study was a cluster randomised controlled trial evaluating the feasibility and acceptability of the intervention [45]. A study by Armstrong [46] evaluated the impact of a common factors approach in training the LHWs. Rahman [47] conducted a multi-method study to investigate the challenges and opportunities in developing an intervention for perinatal depression. Finally, Maulik et al. [48] conducted a study to develop and test a tool for referral and treatment as well as gather research to understand perceptions about mental health in rural India.

As per inclusion criteria, studies only included participants with anxiety, depression or CMDs, however one study included participants with mild to severe psychosocial problems and in one study the mental health condition was unknown.

In three studies the intervention being delivered was counselling, two used the Thinking Healthy Programme based on CBT techniques, one study focused on psychoeducation and another used a form of social support. One study used Problem Solving Therapy (PST) whilst another used a combination of PST and behavioural activation (BA) which was given the term problem management plus (PM+). Lastly, another study trained LHWs in an intervention that utilised a mobile technology based electronic decision support system (EDSS) to improve the identification and management of individuals.

### Characteristics of LHWs and the training received

A description of the characteristics of the LHWs is presented in Table 2. LHWs trained to deliver the interventions included two studies using paraprofessional counsellors, one study using lady health workers and the remaining four studies using lay persons. Two studies used volunteers, one in the form of volunteer befrienders and the other in the form of peer volunteers (PVs). Finally, one study used accredited social health activists (ASHAs) to deliver the psychological intervention.

**Table 2:** Table of characteristics of LHWs

First author/year	LHW	Total no. of LHWs	Previous qualifications of LHW	Previous training/role within the community
Armstrong, J., 2003	Paraprofessional counsellors	12	Unknown	Five people had no previous counselling training while seven had some form of training experience
Naeem, S., 2003	Lay women	19	Unknown	No previous training
Jordans, M.J., 2007	Paraprofessionals	26	Minimal educational background (i.e., mainly high school level, with a few college-level participants	Unknown
Rahman, A., 2007	Lady health workers	24	Completed secondary school	Trained to provide mainly preventative mother and child health care and education
Pereira, B., 2011	Lay health counsellor	17	Unknown	Unknown
Coe, C., 2013	Volunteer befrienders	14	Unknown	No previous training
Atif, N., 2016	Peer volunteers	8	They had an education of at least 10 years	No previous training
Maulik, P.K., 2016	Accredited social health activist (ASHA)	4	Unknown	Responsible for providing basic maternal and child care through government funded schemes
Chibanda, D., 2017	Lay health workers	7	Mean of 8 years of education	Previous training in home based care for people living with HIV and AIDS, in community follow-up of persons on TB treatment and in delivering community health education and promotion
Khan, M.N., 2017	Lay helpers	2	Lay helpers had 16 years of education	No previous training

Half of the studies did not report on the previous qualifications of the LHWs, whereas the other half of the studies reported that LHWs had minimal education ranging from 8 to 16 years or had completed high school. Three studies used LHWs that were already working within the community. This included one study in which LHWs had been trained to provide preventative mother and child health care and education and similarly in another study, where LHWs were responsible for providing basic maternal and child care through government funded schemes. In one study LHWs had previous training in home-based care for people living with HIV/AIDS, in community follow-up of persons on TB treatment and in delivering community health education and promotion. In another study using twelve paraprofessional counsellors whilst five had no counselling experience, seven had previously attended some form of training. In four studies, LHWs had no previous training, whilst in two; previous training or role within the community was not documented.

In two studies, a member of the research team delivered the training to the LHWs, whereas in one study the training was delivered by a team of experienced mental health professionals including two clinical psychologists, a general nurse trained in systemic counselling and a psychiatrist. Two studies used a train the trainer approach in which the trainer received training and supervision themselves before training LHWs. However, half of the studies did not document who delivered the training to the LHWs.

Training ranged from 2 days to months and consisted of a variation of lectures, role play and field work. The content of the training in almost all studies mostly focused on understanding the relevant mental health disorder, its management, counselling and communication skills. In three studies, supervision was conducted by a member of the research team, two studies used non-specialist supervisors whilst three studies used mental health professionals. However, supervision was poorly documented with two studies not detailing who conducted supervision and almost all studies not reporting on the format and content of the supervision. Of the two studies that did, supervision involved LHWs sharing their experiences and receiving guidance and support in difficult cases.

### LHWs’ own experiences of receiving training

#### LHWs’ views on training and supervision

Generally, LHWs felt that there was insufficient information given during training and not enough time allowed for training regarding mental health issues [46]. With one LHW also stating that training days were too long, making it difficult to take in all the information provided.

*“There was too much going on* – *I felt I wanted to go and sort it out before the next point.”* (Paraprofessional counsellor) [46, p. 275]

Others expressed a need for more advanced training, with authors commenting that LHWs voiced a need for more opportunities to share and network with other counsellors [43]. Similarly, Atif et al. [41] found that LHWs expressed satisfaction with their training, however believed that more detailed training would be required for dealing with a population with a range of health issues.

*“While most PVs [peer volunteer] found the training sufficient to prepare them for the volunteering role, some felt that a longer and comprehensive training would have equipped them better to deal with the diverse health issues of their target population.”* [41, p. 8]

Satisfaction in training and support was also reported in Maulik et al. [48] and by Coe and Barlow [42]. Armstrong [46] found that participation and engagement in training was facilitated by the LHWs’ views of the learning environment as ‘supportive’ and ‘encouraging’. Furthermore, Pereira et al. [44] found that the LHWs cited *“a comprehensive training program focusing on skills*-*based learning”* as critically important for successful delivery of the intervention [44, p. 8]

*“I liked the training… the trainers were friendly… the way they explained the content was very good. I didn’t experience any problems in understanding it.”* (PV) [41, p. 6]

Most authors found that LHWs felt that supervision enhanced their skills and prepared them for any challenges and issues they faced in delivering the therapy [41, 44], which in one study also included managing their emotional well-being [45].

*“The majority of LHCs [lay health counsellor] reported that experience in the clinics, training before the program and monthly peer group supervision during the program…gave them confidence to overcome the challenges they faced.”* [44, p. 5]

Atif et al. [41] described how field supervision, in which supervisors accompanied the LHWs in therapy delivery, improved the LHWs’ credibility within the community and their trustworthiness.

*“When xx (supervisor) accompanied me, mothers took me more seriously and shared their concerns more openly knowing that I have been properly trained and supervised.”* (PV) [41, p. 6]

Furthermore, in Chibanda et al.’s study [39], increase in skills and confidence led to decreases in the rate of referral to the supervisor. Similarly, Maulik et al. [48] described how with increasing confidence the LHWs required less support from the staff after becoming more comfortable using the screening tool.

*“They indicated that over the years the rate of referral to the supervisor had reduced significantly because they now felt confident with difficult clients.”* [39, p. 148]

Pereira et al. [44] also commented on the LHWs’ emphasis on supervision as a critically important element for successful delivery of the intervention, specifically on the use of a structured on the job supervision protocol involving both on site supervision and once a month group supervision. This emphasis on supervision was also highlighted in two other papers [39, 43]. Finally, only Coe and Barlow [42] reported on the satisfaction of the LHWs’ regarding supervision.

*“The training and guidance they received, coupled with ongoing support from the project co*-*ordinator, was valued and praised by the volunteer befrienders.”* [42, p. 26]

### Barriers and facilitators to therapy delivery

#### Barriers to therapy delivery

A few authors reported on barriers faced by the LHWs in delivering the intervention which affected them in their roles as therapists and hindered therapy delivery. In one study, in which LHWs were peer volunteers often from the same village as the participants, the author commented on how the LHWs experienced difficulties, as the participants were hesitant to reveal personal information possibly fearing a breach of confidentiality or judgement from the LHW especially if they were from the same village [41]. Confidentiality issues were also discussed by Khan et al. [45] who observed that participants were reluctant to disclose their problems when the intervention sessions were made up of participants from the same household.

*“If in any session there were two or three participants from the same house that made it difficult to let the participants share their problems.”* (Lay helper) [45, p. 8]

*“One of the challenges that the PVs experienced was some mothers’ reluctance to disclose personal information, especially when both the PV and the mother belonged to the same village.”* [41, p. 8]

Furthermore, barriers to therapy delivery also included a hesitance to seek help due to the stigma attached to mental illness which influenced the LHW’s level of acceptability.

*“She got upset when I told her that the assessment indicated that she has depression. She said that she is not mad and stopped me from coming in when I went for my next visit.”* (PV) [41, p. 6]

Jordans et al. [43] noted that factors such as training, difficult clients and organisational difficulties limited the LHWs’ abilities and affected their competency in delivering the interventions. Furthermore, the author addressed how for some LHWs the extra responsibilities were difficult to carry out with their current duties due to a lack of time and conflicts of interest. Other roles and responsibilities were also reported by Maulik et al. [48] who discussed LHWs’ concerns of the harvest season *“as both ASHAs [accredited social health activists] and many villagers work as season labourers in the fields during harvest.”* [48, p. 7]

*“For some multiple responsibilities were compatible with the counselling activities, but for others they were distracting or even incompatible…due to time restraints or confusing due to conflicts of interest, and generally reflective of a lack of management’s prioritization of psychosocial services.”* [43, p. 63]

#### Use of aids in facilitating therapy delivery

##### Physical aids

The use of materials to aid in the delivery of the intervention was briefly described in Armstrong [46], in which the usefulness of handouts and video demonstrations of counselling sessions was emphasised by the LHW. Similarly, in Pereira et al.’s study [44], as well as using a flip chart to aid in the delivery of the therapy, a patient card was also used to facilitate the LHWs in planning the intervention for the patient.

*“The flip chart was observed to facilitate the psychoeducation by helping the patients understand the content better and acting as a guide to the LHC.”* [44, p. 4]

*“LHC’s reported that the patient card with the screening results aided them in planning the intervention for the patient.”* [44, p. 4]

##### Abstract aids

Some studies also reported on the use of abstract aids such as being local to the community and current roles as health workers that facilitated the LHW in delivering the intervention. For example, Rahman [47] commented on how the LHWs were already providing preventative mother and child health care and education and therefore found it easier to understand the intervention. Furthermore, infant care was viewed as a ‘mutually agreed agenda’ and so facilitated the therapists when they met challenges within the therapeutic process.

*“Infant care, on the other hand, was seen as a shared responsibility… It also helped [Lady Health Workers] negotiate difficult situations within the therapeutic process by referring back to this common mutually agreed agenda.”* Rahman [47, p. 7]

Chibanda et al. [39] noted how being local to the community was also found to facilitate therapy delivery as this increased the LHWs’ level of trustworthiness.

*“So, because we are known as Ambuya Utano (Lady Health Workers) there is a certain level of trust we get from PLWH [people living with HIV]. We have lived here for more than 20* *years so we are known because with most of them we see each other at the market, we have helped them in the past…”* (LHW) [39, p. 147]

The LHWs’ own experiences were also seen as a facilitator due to a mutual understanding between the therapist and client which aided in therapy delivery.

*“because I (LHW) also live with HIV when I show understanding (empathy), they are grateful and when I share my own experience this helps to further open up their mind.”* (LHW) [39, p. 147]

#### Motivations for training to become a therapist

The motivation of the LHWs was also important in facilitating successful delivery of the intervention. Atif et al. [41] noted how the LHWs’ perceived personal gains which were described as being altruistic, opportunistic or linked to their well-being as factors contributing to their motivation.

*“Several factors were identified which contributed to their motivation such as their own families endorsement to their role and approval from mothers’ families.”* [41, p. 8–9]

Furthermore, family and community endorsement also motivated LHWs to receive training and deliver the intervention.

*“My family is supportive, without their encouragement, I would not have done this work. It would have been really difficult to leave housework and children.”* (PV) [41, p. 6]

### The factors required to effectively train LHWs to deliver psychological interventions

#### Acceptability

##### Confidentiality

A common theme found by authors was that the level of acceptability and successful delivery of the intervention was dependent on the LHWs’ ability to maintain confidentiality. Naeem et al. [40] commented on how initial reluctance from the clients disappeared as trust in the LHW developed. Similarly, Pereira et al. [44] reported how the LHWs learnt to emphasise confidentiality over time which helped the patients become more comfortable in disclosing personal information.

*“In time the LHCs were accepted by the patients and appreciated by the primary care staff due to their polite and friendly nature and also for maintaining confidentiality.”* [44, p. 8]

*“I stressed to every patient that whatever they would confide in me would remain confidential. I felt these would make the patient feel more comfortable with me and they would ventilate their feelings more easily.”* (LHC) [44, p. 9]

##### Local and trustworthy

Khan et al. [45] found that level of acceptability was also closely linked with the trustworthiness of the LHW, further suggesting that being local and known in the community might be one factor which contributed to the LHWs being trusted compared to a health professional who would be an outsider and unfamiliar to the participants. Atif et al. [41] observed how being local and trustworthy was an advantage to the LHWs, benefitting them in delivering the intervention, which was also found by Chibanda et al. [39].

*“The PVs level of acceptability was dependent upon a number of key factors, including their personal characteristics (e.g. empathy and trustworthiness), being local and linked to the health system, and the intervention perceived as beneficial.”* [41, p. 7]

*“LHWs successfully facilitated the introduction of lay*-*helpers to the community and invited participants to the sessions. They were ideal hosts for Group PM *+* as they are trusted and respected in the communities, overcoming a barrier to accessing women in need.”* [45, p. 9]

Furthermore, as a relationship built between the LHWs and clients overtime, the LHWs became more trusted and accepted, leading to the clients disclosing personal information [40, 41].

#### Developing a therapeutic relationship

Chibanda et al. [39] observed that the LHWs used their own life experiences to help build a relationship with the clients, as well as using physical gestures to connect with them.

*“because I (LHW) also live with HIV when I show understanding (empathy), they are grateful and when I share my own experience this helps to further open up their mind.”* (LHW) [39, p. 147]

*“Connecting with clients came in several forms from touching the hand and other culturally appropriate parts of the body, offering a tissue paper to a crying client, and praying. It was not unusual for the LHWs to use their own life experiences to help create rapport.”* [39, p. 146]

Empathy was also described by two authors as an important factor in building a relationship between the LHWs and the client and was also linked to their level of acceptability [40, 41].

*“Initial resistance from the clients that gradually disappeared as empathy, trust and confidentiality developed.”* [40, p. 2]

#### Collaborative working with other healthcare professionals

Collaborative working can also be regarded as an important component to becoming a successful therapist as it can provide the therapist with more guidance and support from more experienced professionals. Only two authors discussed this collaborative working with Atif et al. describing the LHWs *“good links with the local health system”* [41, p. 5] and Jordans et al. observing that the counsellors expressed a desire for more *“opportunities to share and network with other counsellors”* [43, p. 3]

*“Nobody knows about us whereas, LHWs (Lay Health Workers) are working for the last 18*-*19* *years. It would be really difficult for the PVs to work without their involvement.”* (PV) [41, p. 6]

#### LHWs’ expected skills

Through analysis of the papers it was found that specific skills are required for the intervention to be successful. Chibanda et al. [39] commented on how the LHWs found it difficult to only deal with one problem at a time and that they often felt pressured from the clients to provide solutions. Therefore, it is important for LHWs to be able to learn the skills to manage more than one issue and have the ability to prioritise the more serious concerns.

*“because we are LHWs they think we have answers and we should tell them which problem to start with, so it can take going forward and backward before they identify one problem on their own…”* (LHW) [39, p. 147]

Furthermore, it is necessary for the therapists to have the ability to be able to adapt the intervention to the patient’s needs. Chibanda et al. [39] discussed how the therapists felt that the first session of the intervention required greater emphasis and the subsequent sessions could be shorter. Furthermore, the author noted that as the LHWs realised that clients were not able to attend sessions regularly, adjustments were made to the intervention to ensure that the clients took home a solution that was *“specific, measurable and achievable after the first visit”* [39, p. 149].

*“Ensuring that the bulk of the work was done in the first session was critical because sometimes clients were unable to come back for subsequent sessions. Furthermore, LHWs felt that waiting a week before a problem was reviewed was discouraging for clients.”* [39, p. 146]

Pereira et al. [44] also found that the LHWs were able to find solutions to involve those who were reluctant about the intervention. In contrast, Khan et al. [45] observed that LHWs had difficulties motivating the participants to attend the intervention due to a lack of monetary incentive, therefore it is necessary to provide training to the LHWs to be able to encourage the participants to attend intervention sessions.

*“Participants wanted some monetary incentives and when it was not provided they lost interest and they were not punctual.”* (Lay helper) [44, p. 8]

*“The LHCs observed that providing explanations about the importance of treatment and explaining the mind*–*body link also helped engage patients who were sceptical about the program’s effectiveness.”* [44, p. 5]

It is also important for the therapists to be able to deal with social issues that may arise during the intervention, as often task-shifting approaches are used in LMICs where factors such as financial problems may be a large concern of the patients. Chibanda et al. [39] commented on how problems related to finance were difficult for the LHWs, however over the years and with experience they were able to find solutions by focusing on the reason for needing the money.

*“Some (patients faced) social difficulties like financial problem which is mainly due to seasonal work, daily wages and alcoholism. Another problem was patients not having proper documentation to apply for social schemes e.g. unregistered marriage… But I tried to give them information about various available schemes and how to follow the procedure and some even applied for it.”* (LHC) [44, p. 5]

*“One woman needed $30 for school fees. After we talked about ways of making $30 she came up with several solutions…”* (LHW) [39, p. 148]

### The impact of training and therapy delivery on the LHW

#### LHWs’ gains

##### Impact of training

Some authors commented on the positive benefits that the training had on the LHW. Armstrong [46] noted that the LHW valued the training, emphasising that the stimulating learning environment and working within a group allowed them to share their personal thoughts and feelings and gave them the opportunity to meet new people. Furthermore, the author noted that training led to personal development and a way of enhancing their skills [46], this was a common theme which was also observed by Naeem et al. [40] and Jordans et al. [43].

*“Participants used the training experience as an opportunity to facilitate their development as counsellors, to learn more about themselves, and to increase their personal effectiveness in their day*-*to*-*day lives.”* [46, p. 275]

The training was also found to have a positive impact on the confidence of the LHW [40, 43, 46], with Naeem et al. [40] noting that the training led to a positive approach towards life for the LHWs.

*“I am more self*-*confident in my ability, more self*-*aware and… more open to others views…”* (Paraprofessional counsellor) [46, p. 274]

*“…the training taught us to look for solutions for our problems rather than allowing them to get on our nerves.”* (Lay women) [40, p. 2]

##### Impact of therapy delivery

Most of the authors described the positive impact of delivering the therapy on the LHWs themselves. A common theme that emerged from most papers was that the LHWs developed new skills, which included enhanced listening skills and empathy in Armstrong [46] and learning tolerance and maintaining confidentiality in Naeem et al. [40]. In Maulik et al.’s [48] study, the new skills led to the LHW feeling empowered to talk to the community about mental health, whereas Rahman [47] noted how the skills made them more effective health workers. This benefit to existing duties of the LHWs was also mentioned by Armstrong [46] who suggested that the development of interpersonal skills appeared to be *“helpful in relation to existing human service work.”* [46, p. 274–275]

*“(It) feels like I have opened a part of myself finally, having struggled to find a means to do it for some time*.” (Paraprofessional counsellor) [46, p. 274]

*“It’s just really… I just found it really rewarding. I wanted to give something back to the community really and I feel that I have done that. Um. It’s kind of made me feel accepted in a way.”* (Volunteer befriender) [42, p. 26]

Furthermore, LHWs also gained personal benefit from therapy delivery which involved an improvement in their relationship with others, with Pereira et al. [44] stating that the LHWs used intervention components in handling interpersonal problems. Coe and Barlow [42] also found that LHWs reported an increasing sense of acceptance from therapy delivery, and Naeem et al. [40] found that the LHWs described having an increase in sensitivity and becoming more accepting towards others, as well as understanding the importance of working together and helping each other solve problems.

*“…this training taught us to live our life in a new and different way.”* (Lay women) [40, p. 2]

*“I have gained insight and knowledge on how to approach people sensitively, allow them time… remembering that it is their experience, making no assumptions… that your solution is necessarily theirs.”* (Paraprofessional counsellor) [46, p. 273]

## Discussion

### Summary of the findings

This is the first review of LHWs’ experiences of receiving training to deliver low-intensity psychological interventions. The findings of this review provide support for the feasibility of training non-professionals to deliver psychological interventions as well as highlighting a number of areas that have not been adequately addressed in the published literature, such as how to successfully train and support non-professionals in delivering psychological interventions.

Ten studies were included and ten themes emerged under four overarching areas which were the LHWs’ own experiences of receiving training, barriers and facilitators to therapy delivery, factors required to become a successful therapist and impact of training and therapy delivery on the LHWs were explored in this synthesis.

There are limitations to drawing conclusions about LHWs’ views on training. Limited data about views on the content of training exist and even less on whether the delivery of the training was acceptable. Our findings demonstrate that whilst training is positively received by LHWs, generally it is felt that there is a lack of focus on mental health problems, with more comprehensive training required to support a population with mental health issues. Mental healthcare professionals, in all probability will have existing knowledge about mental illnesses and therapy delivery. In contrast, lay therapists will have little to no knowledge in this area, making delivery of therapy difficult. Despite this, emphasis is placed upon training the therapists in delivering the intervention which although is an important element would be more successful if the therapist had a greater knowledge and broader understanding of the nature and context of mental health issues.

A number of barriers and facilitators to therapy delivery were identified in this review, which researchers should be aware of when planning their own training. A critical barrier highlighted in this review is participants’ hesitance to reveal personal information, fearing a breach of confidentiality. Whilst this barrier is more likely to arise in LMICs where the patient and LHW will often come from the same village [27], and where there is a greater stigma attached to mental health [49]; it is also important to be aware of this when task-shifting interventions in HICs. Given that interventions will often be delivered by a volunteer from within the local area there is a probability that the therapist and patient may know each other or when the intervention is delivered in a group, know fellow group members. Within the wider literature confidentiality and disclosure concerns are a known barrier to accessing mental health services [50, 51]. Loss of confidentiality is closely related to the stigma often surrounding mental health problems within the communities, for example fear of a breach in confidentiality can stem from the fear of stigma and embarrassment of others finding out [52]. The basis of any therapeutic relationship is confidentiality [53] and building trust between individuals, communities and mental health services is important when ensuring access to mental health services [54]. Therefore, those developing and delivering mental health training should carefully consider how best to create a confidential environment which allows patients to safely make personal disclosures. Training should focus on the importance and boundaries of confidentiality, as well as incorporating solutions for when a patient is reluctant to disclose information, or for when disclosure is necessary, for example due to risk of harm to self or others.

Another barrier that was noted in this review was the LHWs’ difficulty in balancing the extra responsibilities of delivering their respective interventions with their current roles and duties. This should be considered when selecting LHWs, particularly in HICs where LHWs will most likely be volunteers who have other work commitments, leading to a lack of time, conflicts of interest and hence affect their competency in delivering the intervention. Moreover, numerous roles and responsibilities may lead to burnout, a phenomenon that is common among the mental health service workforce due to higher workloads and can impact the quality of care provided to mental health consumers [55, 56]. Therefore, preventing burnout through management of workloads and increasing supervision is essential in order to maintain therapists’ satisfaction and a high quality of care [16].

Numerous facilitators were also described in this review, with aids such as training materials guiding the LHWs during intervention delivery. Use of physical materials such as flip charts and handouts not only support the training but can also act as a guide or a point of information for the therapist to refer back to when faced with a challenge. Moreover, abstract factors such as the previous life experiences of LHWs can aid in delivering interventions, as by having similar experiences to the patients they may be more able to empathise with them as well as understand the intervention better [57]. This is a crucial element when selecting LHWs to train, for example, past service users can use their own past experiences and offer guidance through experiential knowledge of mental illness [58], acting as role-models and restoring hope for the patient [59]. Therapist self-disclosure in which the therapist discloses personal information regarding the therapist’s life outside the therapeutic encounter can have facilitating effects on the therapeutic relationship, by building rapport and adding to client comfort [60]. This past experience may also play as motivation for helping others, with the review identifying motivation as a facilitating factor for successful delivery of the intervention. However, qualitative data by Atif et al. [41] shows that despite having well-trained and motivated therapists, it is possible an intervention may not be accepted by the community if the therapists selected are not desirable, or unmatched to the community they are serving. Therefore, in addition to similar experiences of mental health problems it may be useful to recruit LHWs who are peers from the same socio-demographic area as those that they will work with, to ensure they are not perceived as ‘foreign’ by the community. Peers who are persons who share socio-demographic characteristics with the target population, have been used to perform a variety of tasks including counselling, coaching and advocacy [61], with evidence suggesting that peers may have a small additional impact on patient outcomes compared to standard psychiatric care [57].

Training and therapy delivery can lead to multiple gains for LHWs as outlined by this review, which can be used as an incentive by researchers when recruiting people to deliver the intervention. Training can lead to a positive impact in terms of confidence, development of new skills, and provides the opportunity to meet new people. Furthermore, delivery of the intervention can develop the LHWs’ communication skills and lead to an improvement in their relationship with others. In addition, the skills learnt can benefit the individuals in their existing work, especially if they are involved in healthcare and human services work. The benefits of delivering therapy is supported by McLeod [62] who suggests that this type of work is greatly satisfying and individuals feel a privilege to be a part of a process in which someone turns their life around. Moreover, evidence has supported the benefits of incorporating self-practice into training, in which trainees practice therapy techniques on themselves and reflect on their experiences [63]. Therefore, training should include aspects of self-reflection as this can lead to increased empathy for the client and enhance therapeutic understanding and therapist skills [63, 64].

Supervision data is relatively absent from the literature. However, the data available highlights the importance of supervision for successful task-shifting. Supervision is an essential factor for increasing the confidence of the LHW which in turn can lead to less support required as skills are developed. Furthermore, supervision can improve the trustworthiness of the therapist within the community which is of particular importance in LMICs where there is a greater reluctance to seek help for mental health issues and disclose personal information. Clinical supervision is an integral part of psychotherapy training and continuous development, and its importance is supported through empirical evidence suggesting that supervision has positive effects on the trainees’ therapeutic development and competencies [65]. Furthermore, specific supervision formats such as video monitoring and feedback may be effective in improving both therapist competence and treatment outcomes [66].

A number of key messages have been derived out of this review which can help in further improving the quality of training programmes and highlighting the benefits that are available for the therapists. Firstly, duration and skill development should be reconsidered in training programmes to include sufficient time for learning about the nature and context of CMDs. Secondly, it should be explained to LHWs that as this is a new role, expectations of their performance are realistic and it is with supervision and time, that they would be able to improve their skills. A reassuring supervisory role by a senior member of the team can help in building therapists’ confidence and trust within the community, thereby facilitating the learning and therapy-delivering process. Moreover, LHWs should be given the opportunity to collaborate with other healthcare professionals as they can offer further guidance and support through their own experiences. Lastly, it is necessary to ensure that LHWs understand the nature and boundaries of therapeutic relationships and that they have the practical knowledge of how to develop them.

### Strengths and limitations

To our knowledge this review is the first of its kind to focus on the experiences of LHWs trained in delivering psychological interventions. Our literature searches were systematic and transparent, but searching for qualitative studies is complex and requires further investigation.

Whilst the main objective of this review was to explore the experiences of LHWs on training and therapy delivery, the papers included in this review were mostly focused on the intervention itself with training only encompassing a small aspect of the papers. Therefore, it was not possible to gain in depth information on each element of training such as format, content and delivery methods. Furthermore, direct quotes of patient experience were limited for extraction and therefore, much of our findings are based on authors’ interpretations of LHWs experiences. Whilst this has provided interesting data which adds considerably to the literature on the training of LHWs, a greater depth of data direct from LHWs would have been desirable.

In order to maximise data available, a range of psychological interventions and mental health conditions were included. While this facilitated increased data for inclusion, it also created limitations for transferability of the findings. Firstly, the content of training for various types of interventions will differ. Interventions such as CBT and PST are more likely to focus on the delivery components of structured interventions, whereas; counselling interventions will focus on the development of therapeutic relationships, engagement of the patient and person-centred approaches. Secondly, studies investigating CMDs would have to include training on a range of mental health conditions compared to those only investigating a single condition such as perinatal depression, leading to information on the mental illnesses being condensed which could likely influence experiences.

It should be noted that eight out of ten papers meeting the inclusion criteria are from LMICs, where LHWs are commonly used as a solution to the health worker shortage. Although differences can be seen between HICs and LMICs in terms of barriers faced by the LHWs in therapy delivery, there are also factors such as confidentiality that were common across all studies, and the themes that arose are universal themes that would be applicable elsewhere.

### Implications for future research

Key lessons learned from this review should be incorporated into a training framework so that future developers of LHW training interventions are aware of the important factors that need to be incorporated in the training plan. Future research should focus on identifying barriers and facilitators to training LHWs. We should seek to identify in-depth accounts of LHWs experience of training, supervision, and therapy delivery. A further review should also be conducted to explore the experiences of trainers and supervisors. Synthesis of the experience of LHWs, trainers, and supervisors can then inform the future development and delivery of training programmes for lay workers. Furthermore, whilst a review investigating the effectiveness of LHWs delivering psychological interventions has been conducted, the authors have noted that the quality of the studies used was low [23]. Therefore, further high-quality research needs to be conducted to better estimate the effect of LHW delivered interventions for the treatment of depression and anxiety.

## Conclusions

Task-shifting psychological interventions to LHWs has been found to be an effective solution to address the health worker shortage and is often seen as less intimidating and stigmatizing than a formal service. Training is an essential component for successful task-shifting and therefore, to be able to develop effective training programmes for these LHWs, their experiences in training and therapy delivery should be considered. This review highlights the important elements that researchers should be aware of when developing their own training programmes. The findings of this review have added to the evidence base of existing knowledge which should assist researchers to develop high quality training based on clinical and research experience.

## Supplementary information

**Additional file 1.** Full search strategy.

## Data Availability

The data used and analysed during the current study are available from the corresponding author on reasonable request.

## Abbreviations

LHWs: lay health workers
CMD: common mental disorders
CBT: cognitive behaviour therapy
IAPT: increasing access to psychological therapies
LMICs: low- and middle-income countries
CASP: critical appraisal skills programme
HICs: high-income countries
PST: problem solving therapy
BA: behavioural activation
PM+: problem management plus
EDSS: electronic decision support system
PVs: peer volunteers
ASHAs: accredited social health activists
LHCs: lay health counsellors

## References

[1] National Institute for Health and Clinical Excellence. Common mental health disorders: identification and pathways to care. London: NICE; 2011. https://www.nice.org.uk/guidance/cg123/evidence/cg123-common-mental-health-disorders-full-guideline3. Accessed Aug 2019. 31877005

[2] World Health Organization. Depression and other common mental disorders: global health estimates. 2017. http://www.who.int/mental_health/management/depression/prevalence_global_health_estimates/en/. Accessed Aug 2019.

[3] Hirschfeld R, Montgomery SA, Keller MB, Kasper S, Schatzberg AF, Möller H-J, et al. Social functioning in depression: a review. J Clin Psychiatry. 2000. 10.4088/jcp.v61n0405 10830147

[4] Kessler RC, Berglund P, Demler O, Jin R, Koretz D, Merikangas KR (2003). The epidemiology of major depressive disorder: results from the National Comorbidity Survey Replication (NCS-R). JAMA.

[5] Bock J-O, Luppa M, Brettschneider C, Riedel-Heller S, Bickel H, Fuchs A (2014). Impact of depression on health care utilization and costs among multimorbid patients—results from the multicare cohort study. PLoS ONE.

[6] Brenes GA (2007). Anxiety, depression, and quality of life in primary care patients. Prim Care Companion J Clin Psychiatry.

[7] Ensinck KT, Schuurman AG, van den Akker M, Metsemakers JF, Kester AD, Knottnerus JA (2002). Is there an increased risk of dying after depression?. Am J Epidemiol.

[8] Barnhofer T, Crane C, Hargus E, Amarasinghe M, Winder R, Williams JMG (2009). Mindfulness-based cognitive therapy as a treatment for chronic depression: a preliminary study. Behav Res Ther.

[9] O’Hara MW, Stuart S, Gorman LL, Wenzel A (2000). Efficacy of interpersonal psychotherapy for postpartum depression. Arch Gen Psychiatry.

[10] Orgeta V, Qazi A, Spector A, Orrell M (2015). Psychological treatments for depression and anxiety in dementia and mild cognitive impairment: systematic review and meta-analysis. Br J Psychiatry.

[11] Craigie MA, Rees CS, Marsh A, Nathan P (2008). Mindfulness-based cognitive therapy for generalized anxiety disorder: a preliminary evaluation. Behav Cogn Psychother.

[12] Watts SE, Turnell A, Kladnitski N, Newby JM, Andrews G (2015). Treatment-as-usual (TAU) is anything but usual: a meta-analysis of CBT versus TAU for anxiety and depression. J Affect Disord.

[13] NHS England. NHS Mental Health Implementation Plan 2019/20–2023/24. 2019. https://www.longtermplan.nhs.uk/wp-content/uploads/2019/07/nhs-mental-health-implementation-plan-2019-20-2023-24.pdf. Accessed Aug 2019.

[14] Digital N. Psychological therapies: annual report on the use of IAPT services (England 2017–18). 2018. https://files.digital.nhs.uk/52/D3168F/psych-ther-2017-18-ann-rep.pdf. Accessed Aug 2019.

[15] Digital N. NHS workforce statistics 2018. https://files.digital.nhs.uk/52/D3168F/psych-ther-2017-18-ann-rep.pdf. Accessed Aug 2019.

[16] Westwood S, Morison L, Allt J, Holmes N (2017). Predictors of emotional exhaustion, disengagement and burnout among improving access to psychological therapies (IAPT) practitioners. J Ment Health.

[17] McInnis MG, Merajver SD (2011). Global mental health: global strengths and strategies: task-shifting in a shifting health economy. Asian J Psychiatry..

[18] Lewin S, Dick J, Pond P, Zwarenstein M, Aja GN, van Wyk BE (2005). Lay health workers in primary and community health care. Cochrane Database Syst Rev..

[19] World Health Organization. Task shifting: rational redistribution of tasks among health workforce teams: global recommendations and guidelines. 2007. http://apps.who.int/iris/bitstream/10665/43821/1/9789241596312_eng.pdf. Accessed Aug 2019.

[20] Selke HM, Kimaiyo S, Sidle JE, Vedanthan R, Tierney WM, Shen C (2010). Task-shifting of antiretroviral delivery from health care workers to persons living with HIV/AIDS: clinical outcomes of a community-based program in Kenya. JAIDS J Acquir Immune Defic Syndr.

[21] Kaufman JA, Zeng W, Wang L, Zhang Y (2013). Community-based mental health counseling for children orphaned by AIDS in China. AIDS Care..

[22] Petersen I, Bhana A, Baillie K, Mha PPRPC (2012). The feasibility of adapted group-based interpersonal therapy (IPT) for the treatment of depression by community health workers within the context of task shifting in South Africa. Community Ment Health J..

[23] Van Ginneken N, Tharyan P, Lewin S, Rao GN, Meera SM, Pian J (2013). Non‐specialist health worker interventions for the care of mental, neurological and substance‐abuse disorders in low‐and middle‐income countries. Cochrane Database Syst Rev..

[24] Chowdhary N, Sikander S, Atif N, Singh N, Ahmad I, Fuhr DC (2014). The content and delivery of psychological interventions for perinatal depression by non-specialist health workers in low and middle income countries: a systematic review. Best Pract Res Clin Obstet Gynaecol.

[25] Ali BS, Rahbar MH, Naeem S, Gul A (2003). The effectiveness of counseling on anxiety and depression by minimally trained counselors: a randomized controlled trial. Am J Psychother.

[26] Cooper PJ, Tomlinson M, Swartz L, Landman M, Molteno C, Stein A (2009). Improving quality of mother-infant relationship and infant attachment in socioeconomically deprived community in South Africa: randomised controlled trial. BMJ.

[27] Rahman A, Malik A, Sikander S, Roberts C, Creed F (2008). Cognitive behaviour therapy-based intervention by community health workers for mothers with depression and their infants in rural Pakistan: a cluster-randomised controlled trial. Lancet..

[28] Singla D, Lazarus A, Atif N, Sikander S, Bhatia U, Ahmad I (2014). “Someone like us”: delivering maternal mental health through peers in two South Asian contexts. J Affect Disord.

[29] Mueller Martina (2010). Patient perspectives on receiving CBT. Oxford Guide to Surviving as a CBT Therapist.

[30] McClay C-A, Morrison J, McConnachie A, Williams C (2013). A community-based group-guided self-help intervention for low mood and stress: study protocol for a randomized controlled trial. Trials..

[31] Agyapong VIO, Osei A, McLoughlin DM, McAuliffe E (2015). Task shifting—perception of stake holders about adequacy of training and supervision for community mental health workers in Ghana. Health Policy Plan.

[32] Padmanathan P, De Silva MJ (2013). The acceptability and feasibility of task-sharing for mental healthcare in low and middle income countries: a systematic review. Soc Sci Med.

[33] Hammarberg K, Kirkman M, De Lacey S (2016). Qualitative research methods: when to use them and how to judge them. Hum Reprod.

[34] Noblit GW, Hare RD. Meta-ethnography: synthesizing qualitative studies, vol. 11. 1988.

[35] Centre for Reviews and Dissemination. Systematic reviews: CRD’s guidance for undertaking systematic reviews in health care University of York: NHS Centre for Reviews and Dissemination. 2009. https://www.york.ac.uk/media/crd/Systematic_Reviews.pdf. Accessed Aug 2019.

[36] Pope C, Mays N, Popay J (2007). Synthesising qualitative and quantitative health evidence: a guide to methods: a guide to methods.

[37] Braun V, Clarke V (2006). Using thematic analysis in psychology. Qual Res Psychol.

[38] Moher D, Liberati A, Tetzlaff J, Altman DG, Prisma G (2009). Preferred reporting items for systematic reviews and meta-analyses: the PRISMA statement. PLoS Med.

[39] Chibanda D, Cowan F, Verhey R, Machando D, Abas M, Lund C (2017). Lay health workers’ experience of delivering a problem solving therapy intervention for common mental disorders among people living with HIV: a qualitative study from Zimbabwe. Community Ment Health J.

[40] Naeem S, Ali BS, Mubeen S, Iqbal A (2003). The transformative effect of training in counselling and its application, on the community counsellors themselves. JPMA J Pak Med Assoc.

[41] Atif N, Lovell K, Husain N, Sikander S, Patel V, Rahman A (2016). Barefoot therapists: barriers and facilitators to delivering maternal mental health care through peer volunteers in Pakistan: a qualitative study. Int J Ment Health Syst.

[42] Coe C, Barlow J (2013). Supporting women with perinatal mental health problems: the role of the voluntary sector. Community Practitioner..

[43] Jordans MJ, Keen AS, Pradhan H, Tol WA (2007). Psychosocial counselling in Nepal: perspectives of counsellors and beneficiaries. Int J Adv Couns.

[44] Pereira B, Andrew G, Pednekar S, Kirkwood BR, Patel V (2011). The integration of the treatment for common mental disorders in primary care: experiences of health care providers in the MANAS trial in Goa, India. Int J Ment Health Syst.

[45] Khan MN, Hamdani SU, Chiumento A, Dawson K, Bryant RA, Sijbrandij M (2019). Evaluating feasibility and acceptability of a group WHO trans-diagnostic intervention for women with common mental disorders in rural Pakistan: a cluster randomised controlled feasibility trial. Epidemiol Psychiatr Sci..

[46] Armstrong J (2003). Training for paraprofessional counsellors: evaluating the meaning and impact of a common factors approach. Couns Psychother Res.

[47] Rahman A (2007). Challenges and opportunities in developing a psychological intervention for perinatal depression in rural Pakistan—a multi-method study. Arch Women’s Ment Health..

[48] Maulik PK, Tewari A, Devarapalli S, Kallakuri S, Patel A (2016). The Systematic Medical Appraisal, Referral and Treatment (SMART) Mental Health Project: development and testing of electronic decision support system and formative research to understand perceptions about mental health in rural India. PLoS ONE.

[49] Saraceno B, van Ommeren M, Batniji R, Cohen A, Gureje O, Mahoney J (2007). Barriers to improvement of mental health services in low-income and middle-income countries. Lancet..

[50] Gulliver A, Griffiths KM, Christensen H (2010). Perceived barriers and facilitators to mental health help-seeking in young people: a systematic review. BMC Psychiatry..

[51] Salaheddin K, Mason B (2016). Identifying barriers to mental health help-seeking among young adults in the UK: a cross-sectional survey. Br J Gen Pract.

[52] Clement S, Schauman O, Graham T, Maggioni F, Evans-Lacko S, Bezborodovs N (2015). What is the impact of mental health-related stigma on help-seeking? A systematic review of quantitative and qualitative studies. Psychol Med.

[53] MacMurray VD (1986). Confidentiality: the basis of the therapeutic relationship. Issues Relig Psychother.

[54] Memon A, Taylor K, Mohebati LM, Sundin J, Cooper M, Scanlon T, de Visser R (2016). Perceived barriers to accessing mental health services among black and minority ethnic (BME) communities: a qualitative study in southeast England. BMJ Open.

[55] Morse G, Salyers MP, Rollins AL, Monroe-DeVita M, Pfahler C (2012). Burnout in mental health services: a review of the problem and its remediation. Adm Policy Ment Health Ment Health Serv Res.

[56] O’Connor K, Neff DM, Pitman S (2018). Burnout in mental health professionals: a systematic review and meta-analysis of prevalence and determinants. Eur Psychiatry..

[57] Fuhr DC, Salisbury TT, De Silva MJ, Atif N, van Ginneken N, Rahman A (2014). Effectiveness of peer-delivered interventions for severe mental illness and depression on clinical and psychosocial outcomes: a systematic review and meta-analysis. Soc Psychiatry Psychiatr Epidemiol.

[58] Borkman T (1976). Experiential knowledge: a new concept for the analysis of self-help groups. Soc Serv Rev.

[59] Davidson L, Bellamy C, Guy K, Miller R (2012). Peer support among persons with severe mental illnesses: a review of evidence and experience. World Psychiatry..

[60] Audet CT, Everall RD (2010). Therapist self-disclosure and the therapeutic relationship: a phenomenological study from the client perspective. Br J Guid Couns.

[61] Pitt V, Lowe D, Hill S, Prictor M, Hetrick SE, Ryan R, et al. Consumer-providers of care for adult clients of statutory mental health services. 2013. 10.1002/14651858.CD004807.pub2 PMC9750934 23543537

[62] McLeod J. An introduction to counselling. 2013.

[63] Gale C, Schröder T (2014). Experiences of self-practice/self-reflection in cognitive behavioural therapy: a meta-synthesis of qualitative studies. Psychol Psychother Theory Res Pract.

[64] Bennett-Levy J, Turner F, Beaty T, Smith M, Paterson B, Farmer S (2001). The value of self-practice of cognitive therapy techniques and self-reflection in the training of cognitive therapists. Behav Cogn Psychother.

[65] Bambling M, Watkins CE, Milne DL (2014). Creating positive outcomes in clinical supervision. The Wiley international handbook of clinical supervision.

[66] Lambert MJ, Ogles BM, Watkins CE (1997). The effectiveness of psychotherapy supervision. Handbook of psychotherapy supervision.

