# Supplementary material for: The experiences of lay health workers trained in task-shifting psychological interventions: a qualitative systematic review
Source: Int J Ment Health Syst. 2019 Oct 14;13:64. doi: 10.1186/s13033-019-0320-9 (PMC6790996; doi:10.1186/s13033-019-0320-9)
Supplement: Supplementary file 1 — Additional file 1. Full search strategy. [file 13033_2019_320_MOESM1_ESM.docx]

**Ovid Medline search strategy**

1. Allied Health Personnel/
2. Community Health Aides/
3. Nurses Aides/
4. Psychiatric Aides/
5. Caregivers/
6. Voluntary Workers/
7. Community Networks/
8. exp Self-Help Groups/
9. Social Support/
10. Health Manpower/
11. "Personnel Staffing and Scheduling"/
12. (lay adj3 (worker? or visitor? or attendant? or aide or aides or support* or person* or helper? or carer? or caregiver? or care giver? or consultant? or advisor? or counselor? or counsellor? or assistant? or staff)).tw.
13. ((voluntary or volunteer?) adj3 (worker? or visitor? or attendant? or aide or aides or support* or person* or helper? or carer? or caregiver? or care giver? or consultant? or advisor? or counselor? or counsellor? or assistant? or staff)).tw.
14. (untrained adj3 (worker? or visitor? or attendant? or aide or aides or support* or person* or helper? or carer? or caregiver? or care giver? or consultant? or advisor? or counselor? or counsellor? or assistant? or staff or nurse? or doctor? or physician? or therapist?)).tw.
15. (trained adj3 (worker? or visitor? or attendant? or aide or aides or support* or person* or helper? or carer? or caregiver? or care giver? or consultant? or advisor? or counselor? or counsellor? or assistant? or staffor nurse? or doctor? or physician? or therapist?)).tw.
16. (unlicensed adj3 (worker? or visitor? or attendant? or aide or aides or support* or person* or helper? or carer? or caregiver? or care giver? or consultant? or advisor? or counselor? or counsellor? or assistant? or staffor nurse? or doctor? or physician? or therapist?)).tw.
17. ((nonprofessional? or non professional?) adj3 (worker? or visitor? or attendant? or aide or aides or support* or person* or helper? or carer? or caregiver? or care giver? or consultant? or advisor? or counselor? or counsellor? or assistant? or staff)).tw.
18. ((non medical or non health or non healthcare or non health care) adj3 (worker? or visitor? or attendant? or aide or aides or support* or person* or helper? or carer? or caregiver? or care giver? or consultant? or advisor? or counselor? or counsellor? or assistant? or staff)).tw.
19. (community adj3 (worker? or visitor? or attendant? or aide or aides or support* or person* or helper? or carer? or caregiver? or care giver? or consultant? or advisor? or counselor? or counsellor? or assistant? or staff)).tw.
20. (paraprofessional? or paramedic or paramedics or paramedical worker? or paramedical personnel or allied health personnel or allied health worker? or support worker? or non specialist? or specially trained or barefoot doctor? or nurse* aide? or psychiatric aide? or psychiatric attendant? or social worker? or teacher? or school staff or trainer?).tw.
21. (health* adj3 (auxiliary or auxiliaries)).tw.
22. (nurs* adj1 (auxiliary or auxiliaries)).tw.
23. (informal adj (caregiver? or care giver? or carer?)).tw.
24. (self help group? or support group?).tw.
25. ((social or psychosocial) adj (care or support)).tw.
26. (village adj3 worker?).tw.
27. community based.tw.
28. (community adj3 intervention?).tw.
29. community network?.tw.
30. ((health or health care or healthcare) adj manpower).tw.
31. human resources.tw.
32. (task? adj3 shift*).tw.
33. (staff* adj3 chang*).tw.
34. or/1-33
35. ((stress or common mental or depression or anxiety) adj3 disorder*).mp. [mp=title, abstract, heading word, table of contents, key concepts, original title, tests & measures]
36. 34 and 35
37. Grounded Theory/
38. Interview/ or interview*.mp.
39. content analysis.mp.
40. Focus Groups/ or focus group*.mp.
41. discourse*.mp.
42. ethnograph*.mp.
43. phenomenological*.mp.
44. qualitative*.mp. or Qualitative Research/
45. or/37-44
46. 36 and 45
